# Differences in eHealth Access, Use, and Perceived Benefit Between Different Socioeconomic Groups in the Dutch Context: Secondary Cross-Sectional Study

**DOI:** 10.2196/49585

**Published:** 2025-01-07

**Authors:** Lucille Standaar, Lilian van Tuyl, Anita Suijkerbuijk, Anne Brabers, Roland Friele

**Affiliations:** 1 Department of Population Health and Health Services Research Centre for Public Health, Healthcare and Society National Institute for Public Health and the Environment Bilthoven Netherlands; 2 Department Organisation and Quality of Care Netherlands Institute for Health Services Research Utrecht Netherlands; 3 Tranzo Scientific Center for Care and Wellbeing Tilburg University Tilburg Netherlands

**Keywords:** eHealth, digital divide, socioeconomic factors, education, income, neighborhood, health disparities, cross-sectional studies, digital health care, health equity, Netherlands

## Abstract

**Background:**

There is a growing concern that digital health care may exacerbate existing health disparities. Digital health care or eHealth encompasses the digital apps that are used in health care. Differences in access, use, and perceived benefits of digital technology among socioeconomic groups are commonly referred to as the digital divide. Current research shows that people in lower socioeconomic positions (SEPs) use eHealth less frequently.

**Objective:**

This study aims to (1) investigate the association between SEP and eHealth access to, use of, and perceived benefit within the adult Dutch population and (2) evaluate disparities in eHealth access, use, and perceived benefit through three socioeconomic variables—education, standardized income, and the socioeconomic status of the neighborhood.

**Methods:**

A secondary analysis was conducted on data from the Nivel Dutch Health Care Consumer Panel (response rate 57%, 849/1500), to assess access to, use of, and perceived benefits from eHealth. These data were collected to monitor eHealth developments in the Netherlands. eHealth was examined through two concepts: (1) eHealth in general and (2) websites, apps, and wearables. Results were stratified into 9 SEP populations based on 3 indicators—education, standardized income, and socioeconomic status level of the neighborhood. Logistic regression analyses were performed to evaluate whether the outcomes varied significantly across different SEP groups. Age was included as a covariate to control for confounding.

**Results:**

This study confirms the association between eHealth and SEP and shows that low SEP respondents have less access (odds ratio [OR] 5.72, 95% CI 3.06-10.72) and use (OR 4.96, 95% CI 2.66-9.24) of eHealth compared to medium or high SEP respondents. Differences were most profound when stratifying for levels of education.

**Conclusions:**

The access to and use of eHealth has a socioeconomic gradient and emphasizes that SEP indicators cannot be used interchangeably to assess eHealth access and use. The results underline the importance of activities and policies aimed at improving eHealth accessibility and usage among low SEP groups to mitigate disparities in health between different socioeconomic groups.

## Introduction

Digital health care is expected to provide benefits for health care systems, providers, and patients and is considered a solution to address workforce shortages and rising health care costs [1-4]. Moreover, digital health care is anticipated to enhance the quality of care, stimulate patient self-management, and improve health accessibility and equity [1-4]. Digital health care or eHealth is defined as the digital apps that are used in health care. Health care policies focus on increased use of and dependency on the eHealth apps [4-6]. Concerns have been raised that digital health care may not be equally accessible for all [7-13]. Populations with lower socioeconomic positions (SEPs) are more likely to encounter financial, skill, or cognitive barriers to accessing and using eHealth, such as limited access to devices, limited digital health skills, or limited ability to take the initiative in using eHealth [7,11-16]. Additionally, these populations experience challenges in comprehending and implementing health information and healthy behaviors in daily life [17-21].

The demand for health care services is often higher in low-SEP populations, as people with a low SEP endure more often from chronic illnesses [18,19,22,23]. Research shows that low-SEP populations often have different views on health and the possible benefits of healthy behaviors compared to high-SEP populations [24-27]. Studies find that low-SEP populations have less time, more stress, and limited financial capacities to implement healthy behaviors [26]. Next to this, it is theorized that health beliefs often find origin in the health beliefs of previous generations [24-27]. In the context of low SEP, the expectation of a shorter life and the belief that their own behavior has limited influence on their longevity pose barriers to adhering to healthy behaviors [20]. Therefore, the digitalization of health care could seriously impact the access and use of health care for those who need it most [5,6,13,28-31].

National digital connectivity and policies that stimulate the transition toward a digital health care system could improve the implementation and accessibility of eHealth. The European Union and its member states deploy policies to realize the digital transition of health care systems [5,6]. Some states, such as the Netherlands, have been experimenting with digital health care for over a decade [4,6]. Most Dutch households (98%) have fast broadband coverage (2020) and 88% of the Dutch population uses mobile broadband (2019), which indicates the use of a mobile phone or other device with mobile internet access [5]. The level of connectivity in the Netherlands could facilitate the implementation of digital health care [5].

Several countries have developed national eHealth monitoring programs to monitor the uptake and effects of eHealth among health care professionals and citizens [32-34]. From a citizen’s perspective, the monitoring programs focus on the use and evaluation of eHealth that involve citizen interaction [32-34]. This includes applications such as websites, apps, and wearables that citizens can use independently or involve eHealth tools that facilitate digital communication with a health care professional, such as video calls or messaging via patient portals [32-34]. This study is based on secondary data analysis of the Dutch eHealth monitoring program (2021) [29], which collects data about access, use, and perceived benefits from eHealth among Dutch citizens through questionnaires.

There is still limited understanding of the relationship between SEP and eHealth access, use, and perceived benefit and that understanding is generally limited to either access, use, or perceived benefit from eHealth, specific eHealth apps or specific subpopulations. Research showed the relation between SEP and the use of personal health records [35] and mobile apps [36]. Other research focuses on either the benefit from [37] or the use of eHealth [38,39] or specific patient groups such as cancer survivors [40,41], or citizens bound to specific locations [42]. To our knowledge, insight into differences in eHealth access, use, and perceived benefits and how different indicators for SEP display these differences within the Dutch general population aged 18 years and older are largely unknown. This study aims to assess differences in eHealth-related access, usage, and perceived benefits for different socioeconomic populations, based on education, standardized income, and socioeconomic status (SES) level of the neighborhood in the Netherlands. The findings of this study give insight into the disparities in access to, use of, and perceived benefit from eHealth in a highly connected country with an increasingly digitalized health care system. The results are insightful for other contexts that aim for or experience the same ambitions to transition to a digital health care system.

## Methods

### Panel

Data from the Dutch Health Care Consumer Panel (DHCCP) were used [43]. The DHCCP is a panel managed by Nivel (the Netherlands Institute for Health Services Research) and currently (as of September 2023) consists of approximately 11,500 panel members aged 18 years and older [43]. For this study, a study sample of 1.500 panel members was drawn by researchers from DHCCP. The study sample was representative of the Dutch population aged 18 years and older regarding age and sex [43]. Background characteristics of panel members, including their sex, age, level of education, net monthly income per household, and 4-digit postal codes were known. The panel was periodically renewed to ensure representative samples of the adult population in the Netherlands can be drawn. New panel members were recruited by buying an address file from an address supplier [43]. As a result, possible new members were sampled at random from the general population in the Netherlands [43]. The panel could only be joined through invitation. It was not possible for people to sign up on their own initiative [43]. Upon membership, panel members were informed of the purpose, scope, method, and use of the panel [43]. Based on this information, participants could give permission to participate in the panel [43].

### Ethical Considerations

According to the Dutch legislation, neither obtaining informed consent nor approval by a medical ethics committee was obligatory for doing research within the DHCCP [43,44]. Data analysis was conducted with pseudonymized data, according to the privacy regulations of the DHCCP, in compliance with the General Data Protection Regulation [44]. The privacy of the panel members was protected. All data were carefully stored by Nivel [43]. Personal information such as addresses was stored separately from the data of the questionnaires [43]. The privacy of the panel members in the study sample was guaranteed by DHCCP [43]. The researcher (LS) who analyzed the data had no access to the personal information of the panel members [43]. A written or digital informed consent was obtained at the time of registration of a new member to the panel [43]. Panel members were asked to participate approximately 4 or 5 times per year [43]. Participation was voluntary.

### Data Collection

Data on the population’s perspective on eHealth were collected via the DHCCP as part of a larger monitoring study into the perceptions, experiences, and usage of eHealth in the Netherlands [43]. A questionnaire was developed and reviewed by a team of representatives from the health care field in the Netherlands. The questionnaire was based on earlier distributed questionnaires of the monitoring study and was adjusted to reflect market developments [34]. The questionnaire was distributed via email and post (according to the preferences of the panel members) in May 2021. A digital reminder was sent after 1, 2, and 3 weeks after the start of the questionnaire and 1 written reminder was sent after 2 weeks. Panel members had 4 weeks to respond.

### Socioeconomic Position Indicators

#### Overview

The concept of SEP is complex, as it results from the interaction between individual, social, economic, cultural, and societal factors [45,46]. In this study, 3 different operationalizations of SEP were used to study the digital divide in the context of eHealth—education as a historic starting point, standardized household income as a measure of current wealth, and SES level of the neighborhood to include environmental influence [46-49].

#### Education

The education levels were defined as low (none, primary school, or prevocational education); medium (secondary or vocational education level 1, 2, 3, or 4); and high (professional higher education or university) [50].

#### Standardized Income

Standardized income was defined as the net monthly income of the household adjusted for number of household members. The net income was converted to the equivalent of the net income of a single adult household by using equivalence factors from Statistics Netherlands (the Dutch Institute for Population Statistics) [51]. Some respondents acknowledged having children or other adults living in their household apart from their partner or children older than 18 years of age but did not specify the number. In the case of an unknown amount of children, 1.57 children were assumed. In case of an unknown amount of extra adults 1 extra adult was assumed. Assumptions were derived from the averages in Dutch households [50,52,53]. Information gathered from the panel members about their monthly net income was in ranges, and the mean of the range was taken as the monthly net income. Standardized income was divided into three categories (1) low (between €0 and €1659 [US $1718.56] per month; The used conversion rate was applicable on May 1, 2021), (2) medium (between €1660 [US $1719.59] and €2332 [US $2415.72] per month), and (3) high (more than €2332 [US $2415.72] per month). The categories were derived from the quartile distribution of the net income of the Dutch households (2020) [54].

#### Socioeconomic Status Level of the Neighborhood

The SES level of the neighborhood of all respondents was determined using the Social Economic Status-Wealth Education Employment (SES-WOA) score (2019) from the Statistics Netherlands. The SES-WOA score was based on the wealth, educational status, and recent employment history of households in the neighborhood [55,56]. The SES-WOA score was matched to the respondent by the 4-digit postal code. The neighborhoods of the respondents were categorized as (1) low (first tertile of SES score: –0.89 to 0.042), (2) medium (second tertile of SES score: 0.043-0.21), and (3) high (third tertile of SES score: 0.21-0.71). The average score of Dutch neighborhoods was 0.092 (SD 0.23; range –0.89 to 0.71) [55,56].

### eHealth and the Digital Divide

For the interpretation of the data, the digital divide model was used. The digital divide model published by van Dijk et al [57] conceptualized that individuals’ SEPs influence the available resources to access, use, and benefit from new digital media. In this study, the digital media in focus was eHealth.

Two concepts of eHealth were examined: (1) eHealth in general and (2) websites, apps, and wearables. Items in the questionnaire that informed these 2 concepts were matched to the 3 levels of the digital divide model [57], namely level 1—access, level 2—use, and level 3—perceived benefit. Operationalization of the variables measuring access, use, and perceived benefit for eHealth in general and websites, apps, and wearables can be found in Multimedia Appendix 1.

First, eHealth in general was studied to gain insight into an overall interest toward digital apps in health care. The levels of digital divide that were studied are access and the perceived benefit. Access was measured by motivation. Here respondents were asked what their general thoughts are about digital apps used in health care. The perceived benefit was operationalized by measuring to what extent the respondents perceived themselves as making more conscious decisions about their health as a result of eHealth use.

Second, eHealth in terms of websites, apps, and wearables was studied to gain insight into the use of these specific eHealth tools to improve health or provide support in coping with a disease. The digital divide levels that were taken into account are access and use. Access was measured by motivation and physical access. Motivation was measured by asking the respondents if they have used or would like to use websites, apps, and wearables for their health. For physical access, the respondents were asked if they have access to an electronic device with internet.

The digital divide concept use was measured by barriers in use, diversity of use, and frequency of use. For the concept barriers in use, the respondents were asked if they experienced barriers in the use of websites, apps, and wearables. The use of websites, apps, and wearables was further operationalized in 2 variables—diversity of use and frequency of use. For these variables, the respondents were asked about 16 different websites, apps, or wearables if they used the app (once or more than once). Diversity entails the variety of websites, apps, and wearables used, while the frequency of use operationalizes the number of times (more than once) the apps were used.

### Statistical Analysis

Descriptive analyses were used to describe the demographics and the outcome of the variables measuring the access, use, and perceived benefit for eHealth in general and websites, apps, and wearables. The variables measuring access, use, and perceived benefit were constructed by combining items from the original questionnaire. The operationalization of these variables can be found in Multimedia Appendix 1.

The outcomes were stratified by the 3 variables of SEP—educational level, standardized income, and SES level of the neighborhood. The differences in eHealth access and usage between SEP populations were investigated using logistic regression analysis. Ordered logistic regression was used for testing the differences in perceived benefit between SEP populations. Age was included in the analysis to test for confounding, as age is associated with both health and familiarity and use of digital media [10,58,59]. The correlation between the independent variables was determined via the Spearman rank order correlation coefficient. Data analysis was conducted using the Stata Statistical Software release (version 16.1; StataCorp) [60]. A *P*<.05 was considered statistically significant. Variables of physical access and diversity of use were not included in the logistic regression analysis because there were too few cases in outcome categories to meet the assumptions of the logistic regression analysis. The univariate outcomes are presented in Multimedia Appendix 2.

## Results

### Sample Characteristics

In total, 849 panel members responded to the questionnaire, resulting in a response rate of 56.6% (849/1500). Among the panel population of 1500, 8.9% (133/1500), 40% (600/1500), and 48.7% (731/1500) had a low, medium, and high level of education, respectively. Of these groups, 55.6% (74/133), 59.3% (356/600), and 54.3% (397/731) responded to the questionnaire. Regarding standardized income 35.8% (537/1500), 31.8% (477/1500), and 27.9 (419/1500) had low, medium, and high levels of standardized income, respectively. Of these groups, 57.2% (307/537), 56.4% (269/477), and 54.9% (230/269) had responded to the questionnaire. Finally, for SES-level of the neighborhood, 42.8% (642/1500), 32.6% (489/1500), and 23.1% (346/1500) had low, medium, and high levels of SES-level of the neighborhood in the panel population, respectively. Of these groups, 56.1% (360/642), 58.1% (284/489), and 55.2% (191/346) responded to the questionnaire.

The demographics of the study population can be found in Table 1. An overview of the study population stratified by education, standardized income, and SES level of the neighborhood can be found in Multimedia Appendix 3. Overall, the sample contained the same distribution of males or females as in the general population. When stratified for age category, our sample contained slightly more respondents aged 40 years and older and fewer respondents aged 18-39 years as compared to the general population. A frequency table of males and females in 3 age categories from the study population and the general Dutch population can be found in Multimedia Appendix 4 [61]. Compared to the Dutch general population, the study population had more males (11.3% general population, 14.9% study population) and females (13.1% general population, 15.4% study population) in the age category of 65 years and older. In terms of sex, the distribution was equal between both populations (Dutch general population of 49.3% male and 50.7% female, study population male 48.7% and female 51.3%). The mean age was 54 (SD 16.96) years. The lowest number of respondents was in the low educational level subpopulation (74/849, 8.72%) and the highest in the high educational level subpopulation (397/849, 46.76%). A high educational level was significantly and positively associated with a high standardized income level, as indicated by the correlations of educational level and standardized income level: ρ=0.37 (*P*<.001), educational level and SES level of the neighborhood: ρ=0.021 (*P*=.55), and standardized income level and SES level of the neighborhood: ρ=0.035 (*P*=.33) [62].

**Table 1:** Demographic description of the study population (n=849). Study population was sampled (2021) from a representative population (N=1500) of general Dutch population aged 18 years and older.

			Total (n=849)
**Sex, n (%)**			
	Male	413 (48.6)	
	Female	435 (51.2)	
	Missing	1 (0)	
**Age (years)**			
	Mean (SD)	54 (16.96)	
	Range	19-92	
**Average household size**			
	Mean (SD)	2.29 (1.11)	
	Range	1-7	
**Number of households with children younger than 18 years, n (%)**			
	No	604 (71.1)	
	Yes	235 (27.7)	
	Missing	10 (1.2)	
**Number of households with children older than 18 years, n (%)**			
	No	763 (89.9)	
	Yes	76 (9)	
	Missing	10 (1.2)	
**Education**			
	Mean (SD)	2.39 (0.65)	
	Range	1-3	
	Low, n (%)	74 (8.7)	
	Medium, n (%)	356 (41.9)	
	High, n (%)	397 (46.8)	
	Missing, n (%)	22 (2.6)	
**Standardized income**			
	Mean (SD)	1.90 (0.81)	
	Range	1-3	
	Low, n (%)	307 (36.2)	
	Medium, n (%)	269 (31.7)	
	High, n (%)	230 (27.1)	
	Missing, n (%)	43 (5.1)	
**SES^b^ level of the neighborhood**			
	Mean (SD)	1.80 (0.79)	
	Range	1-3	
	Low, n (%)	360 (42.4)	
	Medium, n (%)	284 (33.5)	
	High, n (%)	191 (22.5)	
	Missing, n (%)	14 (1.7)	

^a^Education level—low (none, primary school or prevocational education); medium (secondary or vocational education level 1, 2, 3, or 4); and high (professional higher education or university). Standardized income was divided into 3 categories—low (between €0 and €1659 [US $1718.56] per month); medium (between €1660 [US $1719.59] and €2332 [US $2415.72] per month); and high (more than €2332 [US $2415.72] per month). The SES level of the neighborhood was determined using the Social Economic Status-Wealth Education Employment (SES-WOA) score (2019) from Statistics Netherlands. The SES-WOA score was based on the wealth, educational status, and recent employment history of households in the neighborhood [55,56]. Categories: low (first tertile of SES score: –0.89 to 0.042); medium (second tertile of SES score: 0.043-0.21); and high (third tertile of SES score: 0.21-0.71). Not all values add up to 100% due to missing values.

^b^SES: socioeconomic status.

### Results of the Relation Between the Digital Divide Levels and the Socioeconomic Position Indicators

#### Overview

**Figure 1:**
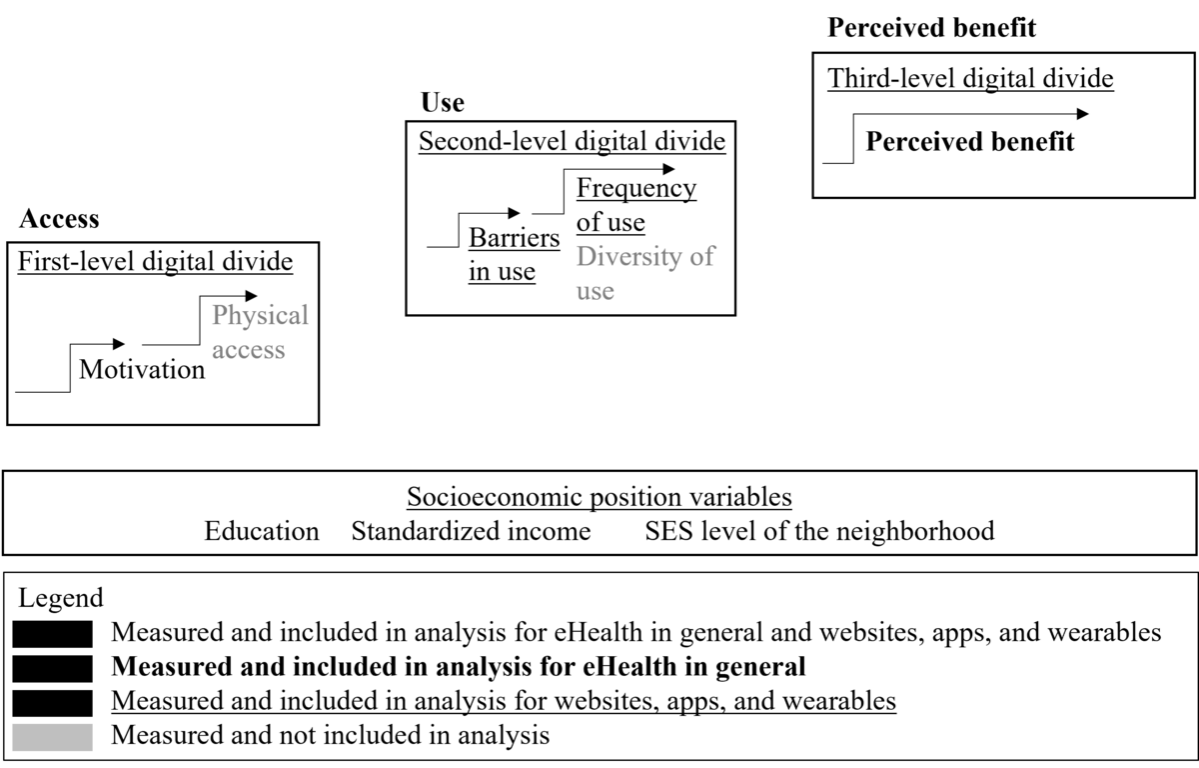
Overview of the concepts of access, use, and perceived benefit, the variables matched to these concepts and the socioeconomic position indicators used in this study. Legend indicates whether the variables were matched for eHealth in general (bold), websites apps and wearables (underlined) or both. Second, the legend indicates whether the variables were viable, either included (black) or not included (grey), for logistic (ordered) regression analysis [57]. SES: socioeconomic status.

**Figure 2:**
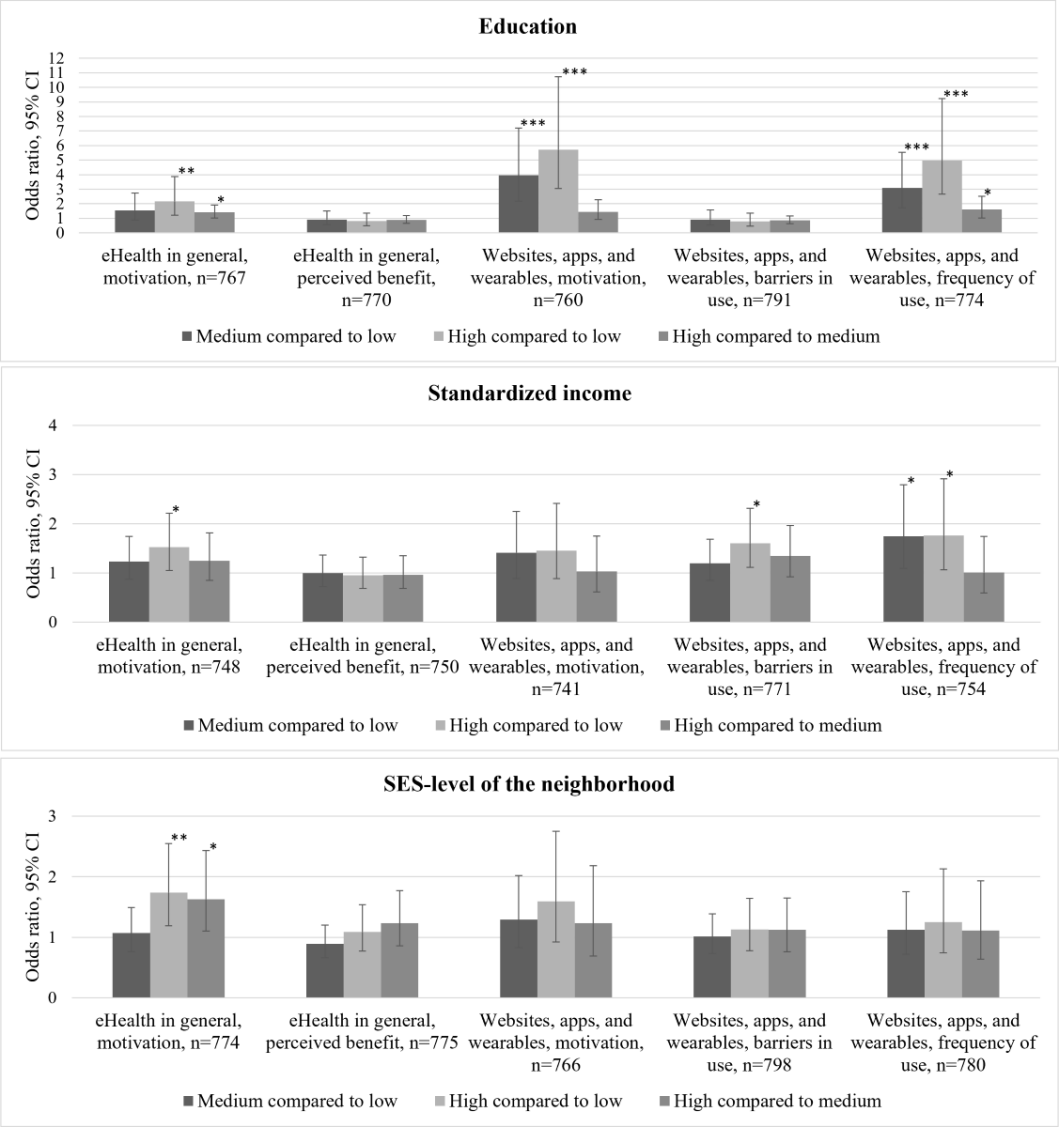
The relation between access, use, and benefit of eHealth and low, medium, and high levels of 3 SEP indicators—education, standardized income, and SES-level of the neighborhood. Results from a questionnaire (2021) answered by a representative study population (n=849) for the general Dutch population aged 18 years and older. Access: motivation, use: barriers in use and frequency of use, and perceived benefit: perceived benefit. For each SEP indicator, a bar graph of the results of logistic (ordered) regression analysis was presented. Each bar shows the odds ratio (OR) and the 95% CI for the difference between SEP levels for each digital divide concept. For each SEP indicator, the comparisons made were medium compared to low (dark grey), high compared to low (light grey), and high compared to medium (medium grey). The number of respondents (n) included in the analysis for each digital divide concept was indicated. The variable barriers in use were recoded to ensure that a positive score (1) reflected the outcome: no experienced barriers. Positive ORs (OR>1) should be interpreted that the primary group in the comparison, in the case of this study either medium or high SEP, had more likelihood of not experiencing barriers in use. *P>.05, **P>.01, ***P>.001. SEP: socioeconomic position; SES: socioeconomic status.

An overview of the measured and analyzed variables can be found in Figure 1. The frequencies of these outcomes can be found in Multimedia Appendix 2. Table 2 presents a descriptive overview of access, use, and perceived benefit, stratified by SEP indicators. The results showed that the outcome differed the most when the population was stratified by education. Figure 2 presents the associations between access; use; perceived benefit; low, medium, and high education; standardized income; and SES level of the neighborhood populations. The underlying data used for Figure 2 is presented in Multimedia Appendix 5.

**Table 2:** Frequency distribution of access, use, and perceived benefit for (1) eHealth in general and (2) websites, apps, and wearables, stratified by education, standardized income, and SES^a^ level of the neighborhood. Frequencies are derived from results of a questionnaire (2021) conducted among a sample of the general Dutch population (N=1500), final study population (n=849)^b,c^.

				Total		Education							Standardized income						SES level of the neighborhood				
						Low		Med^d^		High		Low			Med		High		Low		Med		High
Population, n				849		74		356		397		307			269		230		360		284		191
**eHealth in general**																							
	**Access**—**motivation, n (%)**																						
		No motivation	386 (45.5)		46 (62.2)		177 (49.7)		152 (38.3)		153 (49.8)			124 (46.1)		90 (39.1)		174 (48.3)		141 (49.6)		68 (35.6)	
		Motivation	401 (47.2)		21 (28.4)		153 (43.0)		218 (54.9)		131 (42.7)			127 (47.2)		123 (53.5)		157 (43.6)		127 (44.7)		107 (56)	
	**Perceived benefit—perceived benefit, n (%)**																						
		Totally disagree—disagree	211 (24.9)		17 (23.0)		80 (22.4)		108 (27.2)		70 (22.8)			68 (25.3)		65 (28.3)		85 (23.6)		76 (26.7)		46 (24.1)	
		Not agree nor disagree	370 (43.6)		33 (44.5)		171 (48.0)		159 (40.1)		144 (46.9)			119 (44.2)		89 (38.7)		158 (43.9)		134 (47.2)		75 (39.3)	
		Agree—totally agree	205 (24.1)		17 (23.0)		81 (22.8)		104 (26.2)		69 (22.5)			65 (24.2)		61 (26.5)		87 (24.2)		61 (21.5)		53 (27.7)	
**Websites, apps, and wearables**																							
	**Access—motivation, n (%)**																						
		No motivation	143 (16.8)		35 (47.3)		62 (17.4)		41 (10.3)		59 (19.2)			43 (16.0)		34 (14.8)		65 (18.1)		50 (17.6)		23 (12.0)	
		Motivation	636 (75.0)		29 (39.2)		265 (74.4)		328 (82.6)		221 (72.0)			206 (76.6)		178 (77.4)		263 (73.1)		215 (75.7)		150 (78.5)	
	**Use—barriers in use, n (%)**																						
		Experienced barriers	339 (40.0)		29 (39.2)		140 (39.3)		159 (40.1)		135 (44.0)			109 (40.5)		76 (33.0)		145 (40.3)		119 (41.9)		71 (37.2)	
		No experienced barriers	472 (55.6)		39 (52.7)		201 (56.5)		223 (56.2)		157 (51.1)			149 (55.4)		145 (63.0)		197 (54.7)		156 (54.9)		110 (57.6)	
	**Use—frequency of use, n (%)**																						
		No frequent use	131 (15.4)		29 (39.2)		60 (16.9)		38 (9.6)		59 (19.2)			36 (13.4)		29 (12.6)		57 (15.8)		47 (16.5)		24 (12.6)	
		Frequent use	662 (78.0)		34 (45.9)		274 (77.0)		339 (85.4)		222 (72.3)			219 (81.4)		189 (82.2)		277 (76.9)		226 (79.6)		149 (78.0)	

^a^SES: socioeconomic status.

^b^The variables physical access and diversity of use for websites, apps, and wearables were not presented in Table 2 because these variables could not meet the assumptions for logistic regression analysis. The frequencies of these outcomes can be found in Multimedia Appendix 2.

^c^Not all values add up to 100% due to missing values.

^d^Med: medium level.

#### Access and Motivation

For eHealth in general, as well as for websites, apps and wearables, differences in motivation were found between different levels of education. Differences in motivation were most profound between low or medium versus highly educated respondents in both eHealth in general (odds ratio [OR] 2.18, 95% CI 1.22-3.88) and websites, apps, and wearables (OR 5.72, 95% CI 3.06-10.72). Regarding standardized income, a difference in motivation for eHealth in general was found between high and low standardized incomes (OR 1.52, 95% CI 1.05-2.21). A significant difference in motivation between low (OR 1.74, 95% CI 1.19-2.55) and medium (OR 1.63, 95% CI 1.1-2.43) versus high SES level of the neighborhood was found for eHealth in general.

#### Use, Barriers in Use, and Experienced Barriers

High standardized income was associated with no experience of barriers in use in comparison to low and medium levels of standardized income. This implies that fewer respondents with a high standardized income experienced barriers while using eHealth websites, apps, and wearables (OR 1.60, 95% CI 1.11-2.31). The frequency of eHealth use also differed between respondents with a low, medium, or high level of education, with the most significant difference between high and low educational levels (OR 4.96, 95% CI 2.66-9.24). In terms of standardized income, there were significant differences between low and high (OR 1.76, 95% CI 1.06-2.91) and low and medium (OR 1.74, 95% CI 1.09-2.79) standardized income levels. High SEP respondents were more likely to frequently use an eHealth app, website, or wearable compared to medium or low SEP respondents.

#### Perceived Benefit

There were no significant differences found regarding the perceived benefits between low, medium, or high-SEP populations.

## Discussion

### Principal Findings

This study shows that low SEP respondents have less access to and use of eHealth compared to medium or high SEP respondents. The most significant digital divide observed in this study is related to educational background. The results of this paper contribute on a population level to previous findings that the access and use of eHealth has a socioeconomic gradient. Additionally, the results emphasize that SEP indicators cannot be used interchangeably to assess eHealth access and use.

The results of this study highlight that, across all 3 SEP indicators, the most substantial differences are found in access through motivation. Respondents from higher socioeconomic categories expressed greater motivation to use eHealth, including websites, apps, and wearables, in comparison to those from lower SEPs. Health equity researchers emphasize that comprehending how people perceive health and health care is a complex issue influenced by several societal, contextual, social, and biological individual factors. Therefore, a multidimensional and multicausal approach is necessary to comprehend these disparities [24,63,64]. Weiss et al [28] discuss existing literature and multiple theories as to why differences in eHealth access and use exist between socioeconomic groups. The literature described that the social position of individuals and the influence of the context and organizations surrounding the individual play a role in whether individuals choose to consume digital health care or not [28]. Other literature emphasized that the diffusion of digital health care in society will decrease the digital divide gap as the low-SEP population is assumed to be the latest to adopt [28]. However, the role of health care organizations, social policies, and political decisions that impact individuals’ motivations toward eHealth has not been adequately studied. Current national Dutch policies concerning eHealth are focused on the development and interoperability of eHealth apps, the digital skills of health care professionals and the use of eHealth by older people at home, resulting in eHealth becoming an essential part of the health care system [65]. In countries where health care digitalization is progressing, it would be valuable to examine the potential influence of the government, health care organizations, and businesses on the motivation of low socioeconomic populations toward eHealth. Such research could offer valuable insights into the societal and policy changes required to make eHealth more appealing to low-SEP populations.

In this study, use was examined by studying the barriers in use and frequency of use of eHealth websites, apps, and wearables. The results demonstrated that respondents with a high standardized income level infrequently experienced barriers to using eHealth websites, apps, and wearables compared to lower standardized income levels. Previous studies show that highly educated people often have higher health literacy levels and digital skills and are more in contact with the digital world via their education or profession [10,31,59,66-70]. Other studies show that in the development of eHealth new apps are often pilot-tested by highly educated respondents and, therefore, might be more tailored to the needs of highly educated individuals [9,71,72]. In contrast, other studies point out the variety of health behaviors and health care use within SEP groups. De Boer et al [73] show that low SEP groups have more health care costs but that healthy lifestyle behavior such as smoking or being member of a sports club are attributed greatly to the variety in health care use in each socioeconomic group. In the light of eHealth, Agachi et al [74] showed that the user interface and the type of eHealth offered attributes to the use of eHealth between socioeconomic groups. Results revealed that for the same eHealth program, people living in a low socioeconomic neighborhood use the app-based tool more than people living high in a socioeconomic neighborhood. For the web-based version, results show the opposite emphasizing the importance of the user interface and the accessibility to digital devices, as is also theorized in the digital divide model [49,74]. Both De Boer [73] and Agachi [74] show that behavior-related and technical-related factors play a role in the use of health care and eHealth. The results of this study and other studies showed that understanding and creating insight into the existence of and possible solutions for health disparities is dependent on multiple dimensions. Future research into how the socioeconomic gradient in eHealth access and use are associated with other behavioral and technical factors is important to create an in-depth understanding of disparities in eHealth access and use that can inspire research, policy, and practice.

Results pointed out that high education and high standardized income levels were associated with frequent use of websites, apps, and wearables. This is in accordance with previous research, which shows that a high level of education and income is found to be associated with more access to and use of eHealth [12,13,31]. Surprisingly, no difference was found in the perceived benefits (making more conscious decisions in health because of eHealth use) in any of the SEP indicators, although the frequency of use of websites, apps, and wearables was high (78%) and did show significant differences. This implies that respondents with more frequent use of websites, apps, and wearables had the same perceived benefit, namely, making more conscious decisions due to eHealth use, compared to respondents who have not used websites, apps, and wearables once. Evaluation studies show that high SEP respondents have better outcomes from eHealth use than low SEP respondents [10,30,31,59]. The results of this study might indicate that in the real-life context, even though current eHealth apps might be more suitable for highly educated individuals, eHealth is not used appropriately or with similar discipline as in the clinical trial context.

### Strengths and Limitations

The strengths of this study were the use of a large and representative sample of the Dutch population and the use of 3 SEP operationalizations to provide a broad insight into the effects of SEP on the digital divide. This study also has some limitations. Despite a large number of respondents, the skewed distribution of outcomes across SEP levels hampered the performance of multivariable logistic regression analysis [62]. Next to this, the SEP indicators used are focused on social demographic and economic aspects of SEP. Cultural and other social aspects, such as social networks and cultural background, have not been taken into account.

The questionnaire used in the study was not designed to measure all the different aspects of the digital divide model and is a secondary analysis of the data gathered. Although the majority of digital divide levels could be well matched with questionnaire items, data on the second level digital divide for eHealth in general and the third digital divide level for websites, apps, and wearables was lacking. In some cases, with emphasis on the concept barriers in use for websites, apps, and wearables more in-depth insights into the digital divide levels would have been desirable to improve the validation of the findings. Van Dijk [57] provides concepts to further define the digital divide levels. Access, the first level of digital divide, is described to entail the concepts of motivational access and physical access. Use, the second level of digital divide entails the concepts of digital skills and usage. Usage here encompasses both frequency and diversity of digital media use. Perceived benefit, the third level digital divide, is conceptualized by personal outcomes that are a result of the use of digital media [57]. In this study, the concept barriers in use were used instead of digital skills, as experienced barriers are not limited to barriers formed by a lack of digital skills.

Additionally, for the first and the second level digital divide, the technical design and the information and communication technology of eHealth are of importance [57]. The technical design and the information and communication technology imply factors such as accessibility, usability, mobility, quality, and accessibility of internet access and automation (self-learning devices or software tailored to serve the consumer better and automatically) of devices and apps. These factors are important to facilitate adherence and appropriate use of eHealth apps [57]. The questionnaire provided no insight into these factors.

### Conclusions

The results of this study revealed that differences in motivation for eHealth use are most profound between different socioeconomic populations in the Dutch society, in which low-educated people are likely to be disadvantaged. A successful transition toward digital health care is a social issue that is dependent on the motivation to use eHealth and specific apps. It is imperative that future studies within academia and within the health care field focus on the motivations and needs associated with digital health care, specifically for low-SEP populations. Research on the societal changes stemming from the digital health care transition and the technical and design studies of digital care apps in single-intervention studies are both vital for the realization of an inclusive and comprehensive digital health care system. If eHealth takes a predominant role in the Dutch health care system, it might affect access and use of health care for the citizens who need it the most. The results of this study underscore the importance of policies aimed at facilitating and supporting low-SEP populations in the use of eHealth to reduce differences in health.

## Appendix

Multimedia Appendix 1 Operationalization of access, use, and perceived benefit of eHealth in general and websites, apps, and wearables.

Multimedia Appendix 2 Frequency table of the outcomes of access, use, and perceived benefit for eHealth in general and websites, apps, and wearables gathered via a questionnaire (2021).

Multimedia Appendix 3 Demographic description of the study population and the study population stratified by levels of education, standardized income, and SES level of the neighborhood.

Multimedia Appendix 4 Frequency table of males and females aged above 18 in three age categories of the Dutch general population (2021) and the study population (2021).

Multimedia Appendix 5 Results from logistic (ordered) regression analysis.

## Abbreviations

DHCCP: Dutch Health Care Consumer Panel
OR: odds ratio
SEP: socioeconomic position
SES-WOA: Social Economic Status-Wealth Education Employment
SES: socioeconomic status

## Data Availability

The datasets generated and analyzed during this study are available upon reasonable request from Prof Judith D de Jong (j.dejong@nivel.nl), project leader of the Dutch Health Care Consumer Panel, or the panel’s secretary (conusmentenpanel@nivel.nl).
