# Supplementary material for: Differences in eHealth Access, Use, and Perceived Benefit Between Different Socioeconomic Groups in the Dutch Context: Secondary Cross-Sectional Study
Source: JMIR Form Res. 2025 Jan 7;9:e49585. doi: 10.2196/49585 (PMC11751653; doi:10.2196/49585)
Supplement: Multimedia Appendix 1 [file formative_v9i1e49585_app1.docx]

Questions from a questionnaire conducted in 2021 among a sample of the Dutch population (n=1500) were matched to the concepts access, use and perceived benefit. Some of these concepts were measured by multiple variables. Access: motivation and physical access; perceived benefit: perceived benefit; and use: barriers in use, frequency of use and diversity of use.

| Model stage | Question to respondents | Answer categories | Recoding of answers |
| --- | --- | --- | --- |
| **eHealth in general** | | | |
| Access – Motivation | What are your general thoughts about digital applications in healthcare? | 1. I’m very enthusiastic 2. I would like to try 3. I have to work with it because it’s best for my health 4. I have to work with it because it’s part of the modern times we live in 5. I’m contemplating about what is best for me 6. I am reluctant 7. I do not see added value 8. I am very negative | - Negative motivation (0) if answer category 5, 6, 7 or 8 was checked - Positive motivation (1) if answer category 1,2,3 or 4 was checked |
| Perceived benefit – Perceived benefit | Digital applications in healthcare can lead to me making more conscious decisions for my health | 1. Totally disagree 2. Disagree 3. Neutral 4. Agree 5. Totally agree | - Totally disagree – disagree (1) if answer category 1 or 2 was checked - Neutral (2) if answer category 3 was checked - Agree – totally agree (3) if answer category 4 or 5 was checked |
| **Websites, apps and wearables** | | | |
| Access – Motivation | In the last twelve months I have:  1. Used an app for my health or treatment  2. Used a wearable (such as a smartwatch) for my health or treatment  3. Visited a website for my health or treatment | For each question:   1. Yes, once 2. Yes, often 3. No. and I don’t want to 4. No, and I don’t know if I want to 5. No, but I would want to | - Negative motivation for eHealth applications (0) if for each question answer category 3 or 4 were checked. - Positive motivation for one out of three eHealth applications (1) if for one question the answer category 1, 2 or 5 was checked |
| Access – Physical access | Do you possess a computer, phone or tablet with internet? | 1. Yes 2. No | - No physical access (0) if answer category 2 was checked - Physical access (1) if answer category 1 was checked |
| Use – Barriers in use | While using websites, apps and/or wearables I experience: | 1. No constraining factors 2. Constraining factors | - Barriers in use (0) if answer category 2 was checked - No barriers in use (1) if answer category 1 was checked |
| Use – Diversity of use and frequency of use | In the last twelve months I have:   1. Searched for information via websites or apps about:    1. A disease or treatment    2. The corona virus    3. If I should go to the doctor with a certain problem    4. Lifestyle such as nutrition, physical exercise and/or mental health 2. Tracked data by myself via websites or apps:    1. On my doctors’ visits or treatments    2. On my nutrition or diet    3. On my physical exercise (such as a pedometer) 3. Followed (anonymously) a treatment via a website or apps    1. To quit an addiction such as smoking, alcohol or drug abuse    2. For treatment of psychological problems such as stress management or sleep improvement    3. To learn how to apply healthy/healthier behavior    4. To learn how to manage the consequences of a disease 4. Used equipment that regularly measures health data such as blood pressure or glucose levels. 5. Used an automatic reminder to take my medication via my phone or tablet. 6. Played a computer game to    1. To learn how to apply healthy/healthier behavior    2. To learn how to manage the consequences of a disease 7. Watched online instruction video’s (for example via YouTube) that help me with my nutrition, physical exercise or (mental) health. | For each question:   1. Yes, once 2. Yes, often 3. No. and I don’t want to 4. No, and I don’t know if I want to 5. No, but I would want to | Diversity of use:   - No use of any type of websites, apps and wearables (0) if for all the questions answer category 3, 4 or 5 were checked - Use of 1 or more websites, apps and wearables (1) if for one of the questions answer category 1 or 2 was checked   Frequency of use:   - No frequent use of any type of websites, apps and wearables (0) if for all the questions answer category 1, 3, 4 or 5 were checked - Frequent use of one or more websites, apps and wearables (1) if for one of the questions answer category 2 was checked |
