# Supplementary material for: Differences in eHealth Access, Use, and Perceived Benefit Between Different Socioeconomic Groups in the Dutch Context: Secondary Cross-Sectional Study
Source: JMIR Form Res. 2025 Jan 7;9:e49585. doi: 10.2196/49585 (PMC11751653; doi:10.2196/49585)
Supplement: Multimedia Appendix 2 [file formative_v9i1e49585_app2.docx]

Questionnaire was conducted among a study population (n=849) drawn from a representative population (n=1500) of the general Dutch population aged 18 and above. Access: motivation and physical access; perceived benefit: perceived benefit; and use: barriers in use, frequency of use and diversity of use. Outcomes were stratified by education, standardized income an SES level of the neighborhood. Education level: low (none, primary school or pre-vocational education) ; medium (secondary or vocational education level 1, 2, 3 or 4) (2); and, high (professional higher education or university) (3). Standardized income was divided in three categories, low (between 0 – 1659 € per month) (1); nedium (between 1660 – 2332 € per month) (2); and high (more than 2332 € per month). The SES level of the neighborhood was determined using the SES-WOA score (2019) from Statistics Netherlands. The SES-WOA score was based on the wealth, the educational status and the recent employment history of households in the neighborhood [55,56]. Categories: low (first tertile of SES score [-0.89 – 0.042]) (1); medium (second tertile of SES score [0.043 – 0.21]) (2); and high (third tertile of SES score [0.21 – 0.71]) (3). The variables are constructed from items from the original questionnaire. Operationalization of variables can be found in Multimedia Appendix 1.^[[1]](#footnote-1)^

|  | | Total | Education | | | Standardized income | | | | SES level of the neighborhood | | |
| --- | --- | --- | --- | --- | --- | --- | --- | --- | --- | --- | --- | --- |
|  | |  | Low | Medium | High | Low | Medium | High | Low | | Medium | High |
| **Population (n)** | | 849 | 74 | 356 | 397 | 307 | 269 | 230 | 360 | | 284 | 191 |
| **eHealth in general** | | | | | | | | | | | | |
| **Access** – **Motivation, n (%)** | | | | | | | | | | | | |
|  | No motivation | 386 (45.5) | 46 (62.2) | 177 (49.7) | 152 (38.3) | 153 (49.8) | 124 (46.1) | 90 (39.1) | 174 (48.3) | | 141 (49.6) | 68 (35.6) |
|  | Motivation | 401 (47.2) | 21 (28.4) | 153 (43) | 218 (54.9) | 131 (42.7) | 127 (47.2) | 123 (53.5) | 157 (43.6) | | 127 (44.7) | 107 (56) |
| **Benefit – Perceived benefit, n (%)** | | | | | | | | | | | | |
|  | Totally disagree – disagree | 211 (24.9) | 17 (23) | 80 (22.4) | 108 (27.2) | 70 (22.8) | 68 (25.3) | 65 (28.3) | 85 (23.6) | | 76 (26.7) | 46 (24.1) |
|  | Not agree nor disagree | 370 (43.6) | 33 (44.5) | 171 (48) | 159 (40.1) | 144 (46.9) | 119 (44.2) | 89 (38.7) | 158 (43.9) | | 134 (47.2) | 75 (39,3) |
|  | Agree - totally agree | 205 (24.1) | 17 (23) | 81 (22.8) | 104 (26.2) | 69 (22.5) | 65 (24.2) | 61 (26.5) | 87 (24.2) | | 61 (21.5) | 53 (27.7) |
| **Websites, apps and wearables** | | | | | | | | | | | | |
| **Access – Physical access, n (%)** | | | | | | | | | | | | |
|  | No | 29 (3.4) | 13 (17.6) | 11 (3.1) | 3 (1) | 19 (6.2) | 6 (2.2) | 2 (1) | 10 (2.8) | | 13 (4.6) | 5 (2.6) |
|  | Yes | 816 (96.1) | 61 (82.4) | 342 (96.1) | 393 (99) | 287 (93.5) | 260 (96.7) | 228 (99.1) | 347 (96.4) | | 270 (95.1) | 186 (97.4) |
| **Access – Motivation, n (%)** | | | | | | | | | | | | |
|  | No motivation | 143 (16.8) | 35 (47.3) | 62 (17.4) | 41 (10.3) | 59 (19.2) | 43 (16) | 34 (14.8) | 65 (18.1) | | 50 (17.6) | 23 (12) |
|  | Motivation | 636 (74.9) | 29 (39.2) | 265 (74.4) | 328 (82.6) | 221 (72) | 206 (76.6) | 178 (77.4) | 263 (73.1) | | 215 (75.7) | 150 (78.5) |
| **Use – Barriers in use, n (%)** | | | | | | | | | | | | |
|  | Experienced barriers | 339 (40) | 29 (39.2) | 140 (39.3) | 159 (40.1) | 135 (44) | 109 (40.5) | 76 (33) | 145 (40.3) | | 119 (41.9) | 71 (37.2) |
|  | No experienced barriers | 472 (55.6) | 39 (52.7) | 201 (56.5) | 223 (56.2) | 157 (51.1) | 149 (55.4) | 145 (63) | 197 (54.7) | | 156 (54.9) | 110 (57.6) |
| **Use – Diversity of usage, n (%)** | | | | | | | | | | | | |
|  | No use | 77 (9.1) | 23 (31.1) | 32 (9) | 19 (4.8) | 38 (12.4) | 19 (7.1) | 15 (6.5) | 39 (10.8) | | 25 (8.8) | 11 (5.8) |
|  | Use | 737 (86.8) | 46 (62.1) | 311 (87.4) | 363 (91.4) | 253 (82.4) | 242 (90) | 207 (90) | 307 (85.3) | | 251 (88.4) | 168 (88) |
| **Use – Frequency of use, n (%)** | | | | | | | | | | | | |
|  | No frequent use | 131 (15.4) | 29 (39.2) | 60 (16.9) | 38 (9.6) | 59 (19.2) | 36 (13.4) | 29 (12.6) | 57 (15.8) | | 47 (16.5) | 24 (12.6) |
|  | Frequent use | 662 (78) | 34 (45.9) | 274 (77) | 339 (85.4) | 222 (72.3) | 219 (81.4) | 189 (82.2) | 277 (76.9) | | 226 (79.6) | 149 (78) |

1. Not all percentages add up to a 100% due to rounding or missing values [↑](#footnote-ref-1)
