# Supplementary material for: Differences in eHealth Access, Use, and Perceived Benefit Between Different Socioeconomic Groups in the Dutch Context: Secondary Cross-Sectional Study
Source: JMIR Form Res. 2025 Jan 7;9:e49585. doi: 10.2196/49585 (PMC11751653; doi:10.2196/49585)
Supplement: Multimedia Appendix 3 [file formative_v9i1e49585_app3.docx]

Study population (n=849) was sampled from a representative population (n=1500) of the general Dutch population aged 18 and above (2021). Education level: low (none, primary school or pre-vocational education) ; medium (secondary or vocational education level 1, 2, 3 or 4) (2); and, high (professional higher education or university) (3). Standardized income was divided in three categories, low (between 0 – 1659 € per month) (1); nedium (between 1660 – 2332 € per month) (2); and high (more than 2332 € per month). The SES level of the neighborhood was determined using the SES-WOA score (2019) from Statistics Netherlands. The SES-WOA score was based on the wealth, the educational status and the recent employment history of households in the neighborhood [55,56]. Categories: low (first tertile of SES score [-0.89 – 0.042]) (1); medium (second tertile of SES score [0.043 – 0.21]) (2); and high (third tertile of SES score [0.21 – 0.71]) (3). ^[[1]](#footnote-1),^ ^[[2]](#footnote-2)^

|  | | Total | Education | | | Standardized income | | | | SES level of the neighborhood | | |
| --- | --- | --- | --- | --- | --- | --- | --- | --- | --- | --- | --- | --- |
| Population (n) | |  | Low | Medium | High | Low | Medium | High | Low | | Medium | High |
|  | | 849 | 74 | 356 | 397 | 307 | 269 | 230 | 360 | | 284 | 191 |
| **Sex, n (%)** | | | | | | | | | | | | |
|  | Male | 413 (48.6) | 39 (52.7) | 181 (50.8) | 187 (47.1) | 137 (44.6) | 134 (50) | 123 (53.5) | 184 (51.1) | | 135 (47.5) | 89 (46.6) |
|  | Female | 435 (51.2) | 35 (47.3) | 175 (49.2) | 209 (52.6) | 170 (55.4) | 134 (50) | 107 (46.5) | 175 (48.6) | | 149 (52.5) | 102 (53,4) |
|  | Missing | 1 (0) | 0 (0) | 0 (0) | 1 (0) | 0 (0) | 1 (0) | 0 (0) | 1 (0) | | 0 (0) | 0 (0) |
| **Age, mean (SD, range)** | | 54 (16.96, 19-92) | 65.5 (13.24, 33-87) | 56.4 (15.73, 19-92) | 49.1 (17, 22-89) | 54.4 (16.18, 19-92) | 54.9 (16.29, 25-88) | 51.5 (18.91, 20-89) | 53.2 (17.0, 19-92) | | 55.8 (17.45, 23-88) | 52.1 (16, 20-89) |
| **Average household size, mean (SD, range)** | | 2.29 (1.13, 1-6) | 1.86 (0.96, 1-5) | 2.29 (1.08, 1-7) | 2.38 (1.15, 1-6) | 2.38 (1.27, 1-7) | 2.4 (1.08, 1-5) | 1.98 (0.8, 1-3.57) | 2.01 (1.02, 1-6) | | 2.4 (1.11, 1-6) | 2.67 (1.16, 1-7) |
| **Number of households with children below 18, n (%)** | | | | | | | | | | | | |
|  | No | 604 (71.1) | 65 (87.8) | 259 (72.8) | 268 (67.5) | 212 (69.1) | 180 (66.9) | 195 (84.8) | 292 (81.1) | | 195 (68.7) | 110 (57.6) |
|  | Yes | 235 (27.7) | 8 (10.8) | 96 (27) | 129 (32.5) | 95 (30.9) | 89 (33.1) | 35 (15.2) | 65 (18.1) | | 88 (31) | 80 (41.9) |
|  | Missing | 10 (1.2) | 1 (0) | 1 (0) | 0 (0) | 0 (0) | 0 (0) | 0 (0) | 3 (1) | | 1 (0) | 1 (0) |
| **Number of households with children above 18, n (%)** | | | | | | | | | | | | |
|  | No | 763 (89.9) | 67 (90.1) | 310 (87.1) | 372 (93.7) | 264 (86) | 244 (90.7) | 225 (97.8) | 330 (91.7) | | 257 (90.5) | 168 (88) |
|  | Yes | 76 (9) | 6 (8.1) | 45 (12.6) | 25 (6.3) | 43 (14) | 25 (9.3) | 5 (2.2) | 27 (7.5) | | 26 (9.2) | 22 (11.5) |
|  | Missing | 10 (1.2) | 1 (0) | 1 (0) | 0 (0) | 0 (0) | 0 (0) | 0 (0) | 3 (1) | | 1 (0) | 1 (0) |
| **Education** | | | | | | | | | | | | |
|  | Mean (SD, range) | 2.39 (0.64, 1-3) | 1 (0.00, 1-1) | 2 (0.00, 2-2) | 3 (0.00, 3-3) | 2.12 (0.67, 1-3) | 2.43 (0.6, 1-3) | 2.71 (0.5, 1-3) | 2.39 (0.67, 1-3) | | 2.35 (0.64, 1-3) | 2.46 (0.61, 1-3) |
|  | Low, n (%) | 74 (8.7) | 74 (100) | 0 (0%) | 0 (0%) | 51 (16.6) | 15 (5.6) | 5 (2.2) | 37 (10.3) | | 25 (8.8) | 11 (5.8) |
|  | Medium, n (%) | 356 (41.9) | 0 (0%) | 356 (100) | 0 (0%) | 164 (53.4) | 122 (45.4) | 56 (24.3) | 142 (39.4) | | 132 (46.5) | 79 (41.4) |
|  | High, n (%) | 397 (46.8) | 0 (0%) | 0 (0%) | 397(100) | 88 (28.7) | 129 (48) | 164 (71.3) | 173 (48.1) | | 122 (43) | 97 (51) |
|  | Missing, n (%) | 22 (2.6) | 0 (0) | 0 (0) | 0 (0) | 4 (1.3) | 3 (1.1) | 5 (2.2) | 8 (2.2) | | 5 (1.8) | 4 (2.1) |
| **Standardized income** | | | | | | | | | | | | |
|  | Mean (SD, range) | 2.09 (0.85, 1-3) | 1.35 (0.61, 1-3) | 1.68 (0.74, 1-3) | 2.2 (0.79, 1-3) | 1 (0.00, 1-1) | 2 (0.00, 2-2) | 3 (0.00, 3-3) | 1.88 (0.82, 1-3) | | 1.92 (0.8, 1-3) | 1.93 (0.82, 1-3) |
|  | Low, n (%) | 307 (36.2) | 51 (69) | 164 (46.1) | 88 (22.2) | 307 (100) | 0 (0) | 0 (0) | 140 (38.9) | | 96 (33.8) | 66 (34.6) |
|  | Medium, n (%) | 269 (31.7) | 15 (20.3) | 122 (34.3) | 129 (32.5) | 0 (0) | 269 (100) | 0 (0) | 109 (30.3) | | 96 (33.8) | 61 (31.9) |
|  | High, n (%) | 230 (27.1) | 5 (6.8) | 56 (15.7) | 164 (41.3) | 0 (0) | 0 (0) | 230 (100) | 98 (27.2) | | 75 (26.4) | 56 (29.3) |
|  | Missing, n (%) | 43 (5.1) | 3 (4.1) | 14 (3.9) | 16 (4) | 0 (0) | 0 (0) | 0 (0) | 13 (3.6) | | 17 (6) | 8 (4.2) |
| **SES level of the neighborhood** | | | | | | | | | | | | |
|  | Mean (SD, range) | 1.82 (0.8, 1-3) | 1.66 (0.75, 1-3) | 1.83 (0.78, 1-3) | 1.82 (0.81, 1-3) | 1.78 (0.8, 1-3) | 1.83 (0.79, 1-3) | 1.82 (0.8, 1-3) | 1 (0.00, 1-1) | | 2 (0.00, 2-2) | 3 (0.00, 3-3) |
|  | Low, n (%) | 360 (42.4) | 37 (50) | 142 (39.9) | 173 (43.6) | 140 (45.6) | 109 (40.5) | 98 (42.6) | 360 (100) | | 0 (0) | 0 (0) |
|  | Medium, n (%) | 284 (33.5) | 25 (33.7) | 132 (37.1) | 122 (30.7) | 96 (31.3) | 96 (35.7) | 75 (32.6) | 0 (0) | | 284 (100) | 0 (0) |
|  | High, n (%) | 205 (24.1) | 12 (16.2) | 82 (23) | 102 (25.7) | 71 (23.1) | 64 (23.8) | 57 (24.8) | 0 (0) | | 0 (0) | 191 (100) |
|  | Missing, n (%) | 0 (0) | 0 (0) | 0 (0) | 0 (0) | 0 (0) | 0 (0) | 0 (0) | 0 (0) | | 0 (0) | 0 (0) |

1. Maximum of household members for standardized income high is 3.57, this is not a rounded number because 1.57 child was assumed if respondents had children in their household but the number of children was unknown. [↑](#footnote-ref-1)
2. Not all percentages add up to a 100% due to rounding or missing values [↑](#footnote-ref-2)
