# Supplementary material for: Differences in eHealth Access, Use, and Perceived Benefit Between Different Socioeconomic Groups in the Dutch Context: Secondary Cross-Sectional Study
Source: JMIR Form Res. 2025 Jan 7;9:e49585. doi: 10.2196/49585 (PMC11751653; doi:10.2196/49585)
Supplement: Multimedia Appendix 4 [file formative_v9i1e49585_app4.docx]

Study population (n=849) was sampled from a representative population (n=1500) of the general Dutch population aged 18 and above (2021). M: male; F: female.

|  | | Dutch general population | | Study population | |
| --- | --- | --- | --- | --- | --- |
| **Sexe (%)** | | M(%) | F(%) | M(%) | F(%) |
| **Age category** | | | | | |
|  | 18 - 39 years of age | 17% | 17% | 12% | 13% |
|  | 40 - 64 years of age | 21% | 21% | 22% | 23% |
|  | 65 years of age and older | 11% | 13% | 15% | 15% |
|  | Total | 49% | 51% | 49% | 51% |
