# Supplementary material for: Differences in eHealth Access, Use, and Perceived Benefit Between Different Socioeconomic Groups in the Dutch Context: Secondary Cross-Sectional Study
Source: JMIR Form Res. 2025 Jan 7;9:e49585. doi: 10.2196/49585 (PMC11751653; doi:10.2196/49585)
Supplement: Multimedia Appendix 5 [file formative_v9i1e49585_app5.docx]

The odds-ratio between access, use and perceived benefit from eHealth in general and websites, apps and wearables and the socioeconomic position indicators. Logistic ordered regression analysis was used for perceived benefit. Questionnaire was conducted among a study population (n=849) drawn from a representative population (n=1500) of the general Dutch population aged 18 and above. Access: motivation and physical access; perceived benefit: perceived benefit; and use: barriers in use, frequency of use and diversity of use. Outcomes were stratified by education, standardized income an SES level of the neighborhood. Education level: low (none, primary school or pre-vocational education) ; medium (secondary or vocational education level 1, 2, 3 or 4) (2); and, high (professional higher education or university) (3). Standardized income was divided in three categories, low (between 0 – 1659 € per month) (1); nedium (between 1660 – 2332 € per month) (2); and high (more than 2332 € per month). The SES level of the neighborhood was determined using the SES-WOA score (2019) from Statistics Netherlands. The SES-WOA score was based on the wealth, the educational status and the recent employment history of households in the neighborhood [55,56]. Categories: low (first tertile of SES score [-0.89 – 0.042]) (1); medium (second tertile of SES score [0.043 – 0.21]) (2); and high (third tertile of SES score [0.21 – 0.71]) (3).

|  | | eHealth in general | | | | Websites, apps and wearables | | | | | |
| --- | --- | --- | --- | --- | --- | --- | --- | --- | --- | --- | --- |
|  | | Access | | Perceived benefit | | Access | | Use | | | |
|  | | Motivation | | Perceived benefit | | Motivation | | Barriers in use | | Frequency in use | |
|  | | n | OR  (CI95) | n | OR (CI95) | n | OR (CI95) | n | OR (CI95) | n | OR (CI95) |
| **Education** | | 767 |  | 770 |  | 760 |  | 791 |  | 774 |  |
|  | Medium (comparison population low) |  | 1.55 (0.87 - 2.74) |  | 0.92 (0.56 - 1.50) |  | 3.95 (2.17 - 7.2)  *** |  | 0.92 (0.54 - 1.57) |  | 3.08 (1.72 - 5.54) *** |
|  | High (comparison population low) |  | 2.18 (1.22 -3.88)** |  | 0.81 (0.49 - 1.35) |  | 5.72 (3.06 - 10.72) *** |  | 0.79 (0.46 - 1.36) |  | 4.96 (2.66 - 9.24) *** |
|  | High (comparison population medium) |  | 1.41 (1.03 - 1.92)* |  | 0.89 (0.67 - 1.18) |  | 1.45 (0.92 - 2.27) |  | 0.86 (0.63 - 1.17) |  | 1.61 (1.03 - 2.52)* |
| **Standardized income** | | 748 |  | 750 |  | 741 |  | 771 |  | 754 |  |
|  | Medium (comparison population low) |  | 1.23 (0.87 - 1.74) |  | 0.99 (0.72 - 1.36) |  | 1.41 (0.88 - 2.25) |  | 1.19 (0.85 - 1.68) |  | 1.74 (1.09 - 2.79)* |
|  | High (comparison population low) |  | 1.52 (1.05 - 2.21) * |  | 0.95 (0.68 - 1.32) |  | 1.45 (0.88 - 2.41) |  | 1.60 (1.11 - 2.31) * |  | 1.76 (1.06 - 2.91)* |
|  | High (comparison population medium) |  | 1.24 (0.85 - 1.81) |  | 0.96 (0.68 - 1.35) |  | 1.03 (0.61 -1.75) |  | 1.34 (0.92 - 1.96) |  | 1.01 (0.59 - 1.74) |
| **SES level of the neighborhood** | | 774 |  | 775 |  | 766 |  | 798 |  | 780 |  |
|  | Medium (comparison population low) |  | 1.07 (0.76 -1.49) |  | 0.89 (0.66 - 1.2) |  | 1.29 (0.83 - 2.02) |  | 1.01 (0.73 - 1.39) |  | 1.12 (0.72 - 1.75) |
|  | High (comparison population low) |  | 1.74 (1.19 - 2.55) ** |  | 1.09 (0.77 - 1.54) |  | 1.59 (0.92 - 2.75) |  | 1.13 (0.78 - 1.64) |  | 1.25 (0.74 - 2.13) |
|  | High (comparison population medium) |  | 1.63 (1.1 - 2.43) * |  | 1.23 (0.86 - 1.77) |  | 1.23 (0.69 – 2.18) |  | 1.12 (0.76 - 1.65) |  | 1.11 (0.64 - 1.93) |

^a^Frequencies of the Dutch population were retrieved from Statistics Netherlands [74].

^b^The sex of one participant in the study population was unknown, this participant was excluded from this comparison.

^c^The variables physical access and diversity of use for websites, apps and wearables were not presented because these variables had too few cases in stratified outcome categories to meet the assumptions of the regression analyses.

^d^The logistic regression and ordered logistic regression analyses are corrected with age as a covariate.

* p<0.05, ** p<0.01, *** p<0.001
